# A Rare Presentation of Hepatolithiasis and Acute Cholangitis: A Case Report Highlighting Heterotaxy Syndrome With Left Isomerism

**DOI:** 10.7759/cureus.71069

**Published:** 2024-10-08

**Authors:** Jesse F Simon, Joshua D Katz, David Kosoy, Michael Ashley, Ryan De Melo

**Affiliations:** 1 Radiology, Dr. Kiran C. Patel College of Osteopathic Medicine, Nova Southeastern University, Davie, USA; 2 Radiology, Dr. Kiran C. Patel College of Allopathic Medicine, Nova Southeastern University, Davie, USA; 3 Radiology, Aventura Hospital and Medical Center, Aventura, USA

**Keywords:** acute cholangitis, hepatolithiasis, heterotaxy syndrome, isomerism, polysplenia, situs ambiguous

## Abstract

Heterotaxy syndrome (HS) is a rare congenital disorder characterized by the atypical arrangement of thoracic and abdominal organs. This complex condition is challenging to diagnose due to its ambiguous classification system and the wide spectrum of clinical presentations. HS can be subcategorized into left or right isomerism, with left isomerism typically associated with less severe cardiac malformations but still carrying significant morbidity due to other organ system anomalies. We present the case of a 50-year-old male with a history of diabetes mellitus, hypertension, chronic kidney disease, and cholecystectomy, who presented with worsening abdominal and back pain, fever, and signs of sepsis. Imaging revealed common bile duct thickening and pneumobilia, raising suspicion of acute cholangitis. Further evaluation uncovered multiple congenital anomalies consistent with HS with left isomerism, including congenital malrotation, bilateral hyparterial bronchi, a reversed superior mesenteric artery/vein (SMA/V) relationship, and interruption of the inferior vena cava (IVC) with azygos continuation. The patient was found to have hepatolithiasis complicated by acute cholangitis, leading to septic shock. After aggressive medical and endoscopic intervention, including endoscopic retrograde cholangiopancreatography (ERCP) with lithotripsy, the patient stabilized and was discharged with follow-up care. This case highlights the diagnostic and management challenges associated with HS, particularly when patients present with acute symptoms related to non-cardiac complications. The combination of HS and hepatolithiasis, a rare association, underscores the importance of considering HS in patients with unusual or unexplained anatomical findings. Given the complex nature of this condition, a multidisciplinary approach is crucial for optimizing patient outcomes. HS with left isomerism can present with a wide array of congenital anomalies that may complicate acute medical conditions such as cholangitis. This case illustrates the need for thorough imaging and a high index of suspicion to diagnose HS in adults presenting with atypical abdominal findings. Early recognition and multidisciplinary management are essential in mitigating complications and improving patient outcomes.

## Introduction

Diagnosing heterotaxy syndrome (HS) is challenging for radiologists because of its ambiguous classification system and the overlapping range of clinical presentations. HS, also known as *situs ambiguous*, is defined as the atypical arrangement of thoracic and abdominal organs within the body [1]. The word ‘heterotaxy’ is of Greek origin with *hetero-* meaning ‘different’ and *-taxy* meaning ‘arrangement’ [1-2]. *Situs ambiguous* may share features of *situs solitus* (normal arrangement of viscera) and *situs inversus* (mirror image of viscera) [1]. *Situs inversus* is a rare condition in which all internal organs in the chest and abdominal cavity are in a reversed position including dextrocardia [3].

Patients with HS are further sub-categorized into either left or right isomerism, each defined by their characteristic features. The word ‘isomerism’ is also of Greek origin with iso- meaning ‘equal’ and meros- meaning ‘part’ [1]. Isomerism in the context of HS refers to the mirror image of the atrial appendage and viscera across the left-right axis within the thoracoabdominal cavity. Classic left isomerism or double left-sidedness or polysplenia, implies bilateral bilobed lungs, hyparterial bronchi, bilateral left atria, a central liver, a malpositioned stomach, and multiple small spleens. In contrast, classic right isomerism, or double right-sidedness, or asplenia, implies bilateral trilobed lungs with bilateral minor fissures, eparterial bronchi, bilateral right atria, central liver, and a malpositioned stomach [4]. Adding to the confusion, those with left-isomerism can also share some features of right-isomerism, and vice versa [4,5].

Herein we present the case of a 50-year-old male who presented with worsening abdominal and back pain and was incidentally found to have HS with left isomerism.

## Case presentation

A 50-year-old male with a past medical history of diabetes mellitus, hypertension, chronic kidney disease, and a cholecystectomy over 30 years ago presented to the emergency department (ED) with chronic abdominal and back pain for nine months with significant worsening of symptoms five days prior to presentation. He had not had a bowel movement for three days, felt nauseous, and complained of fever but denied any headache, dizziness, chest pain, dysuria, hematuria, hematochezia, or melena.

In the ED, the patient exhibited tachycardia and hypotension. Abdominal examination revealed diffuse tenderness without rebound or guarding. Initial laboratory results indicated a urinary tract infection (UTI) with positive nitrites, elevated alkaline phosphatase (ALP), aspartate aminotransferase (AST), and bilirubin. Imaging studies, including a right upper quadrant ultrasound and an abdomen CT angiography, revealed common bile duct thickening and pneumobilia raising suspicion for acute cholangitis.

**Figure 1:**
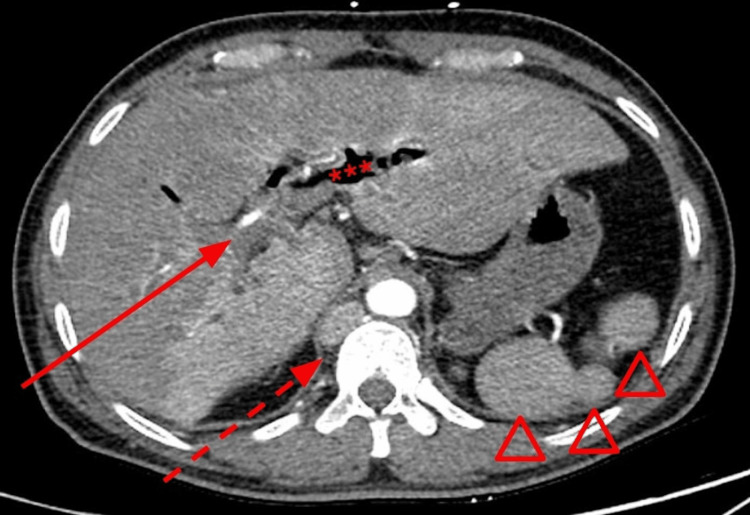
Axial contrast-enhanced CT at presentation demonstrates an intrahepatic biliary ductal dilatation (solid arrow) with pneumobilia (asterisks). There is absence of an intrahepatic IVC (dashed arrow) and multiple splenules indicating polysplenia (hollow arrowheads). CT: Computed tomography; IVC: Inferior vena cava

**Figure 2:**
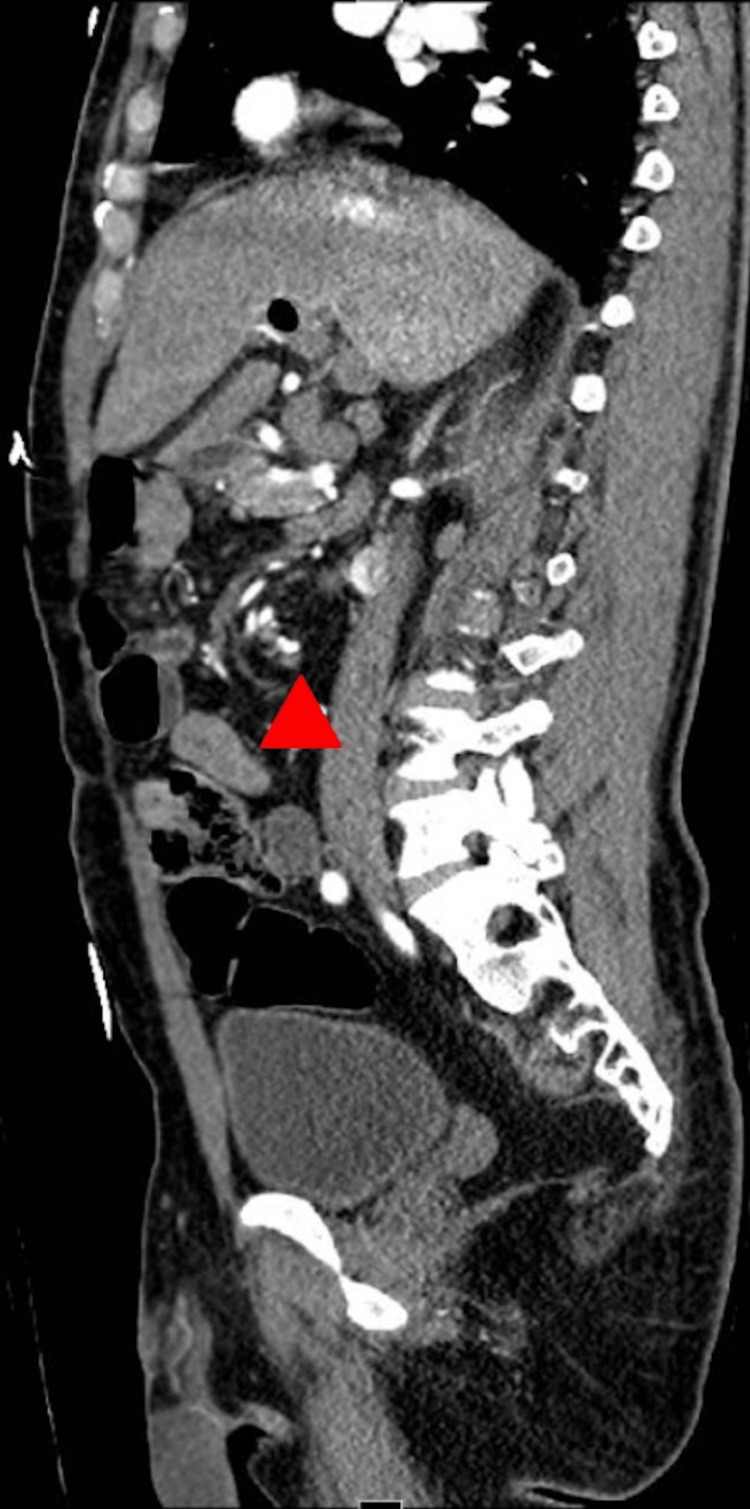
Sagittal contrast-enhanced CT reformat reveals a well-demonstrated presence of gastrointestinal malrotation with swirling of mesenteric vasculature (solid arrowhead). CT: Computed tomography

**Figure 3:**
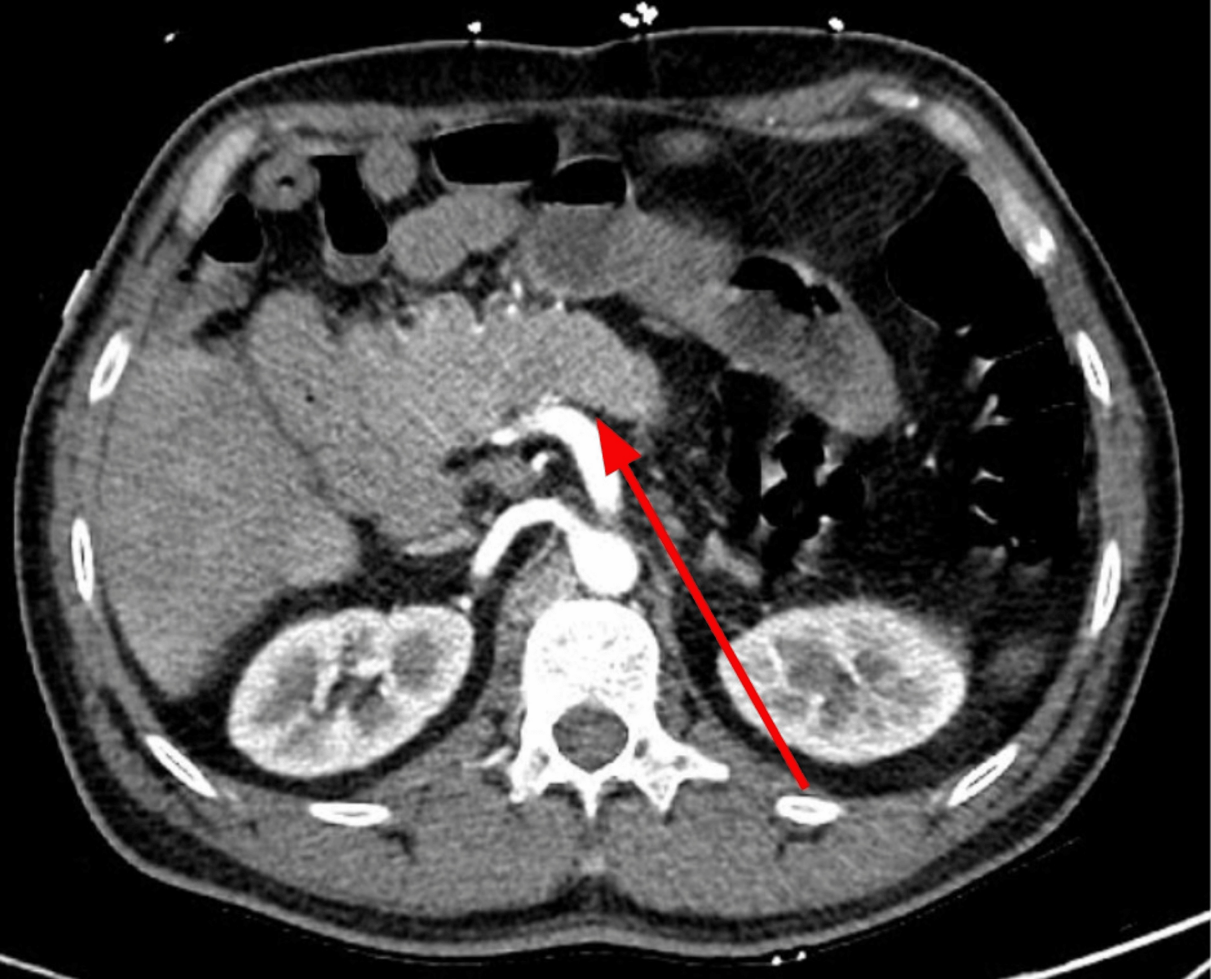
Axial contrast-enhanced CT images at the level of the pancreas demonstrates a truncated dorsal pancreas (solid arrow). CT: Computed tomography

**Figure 4:**
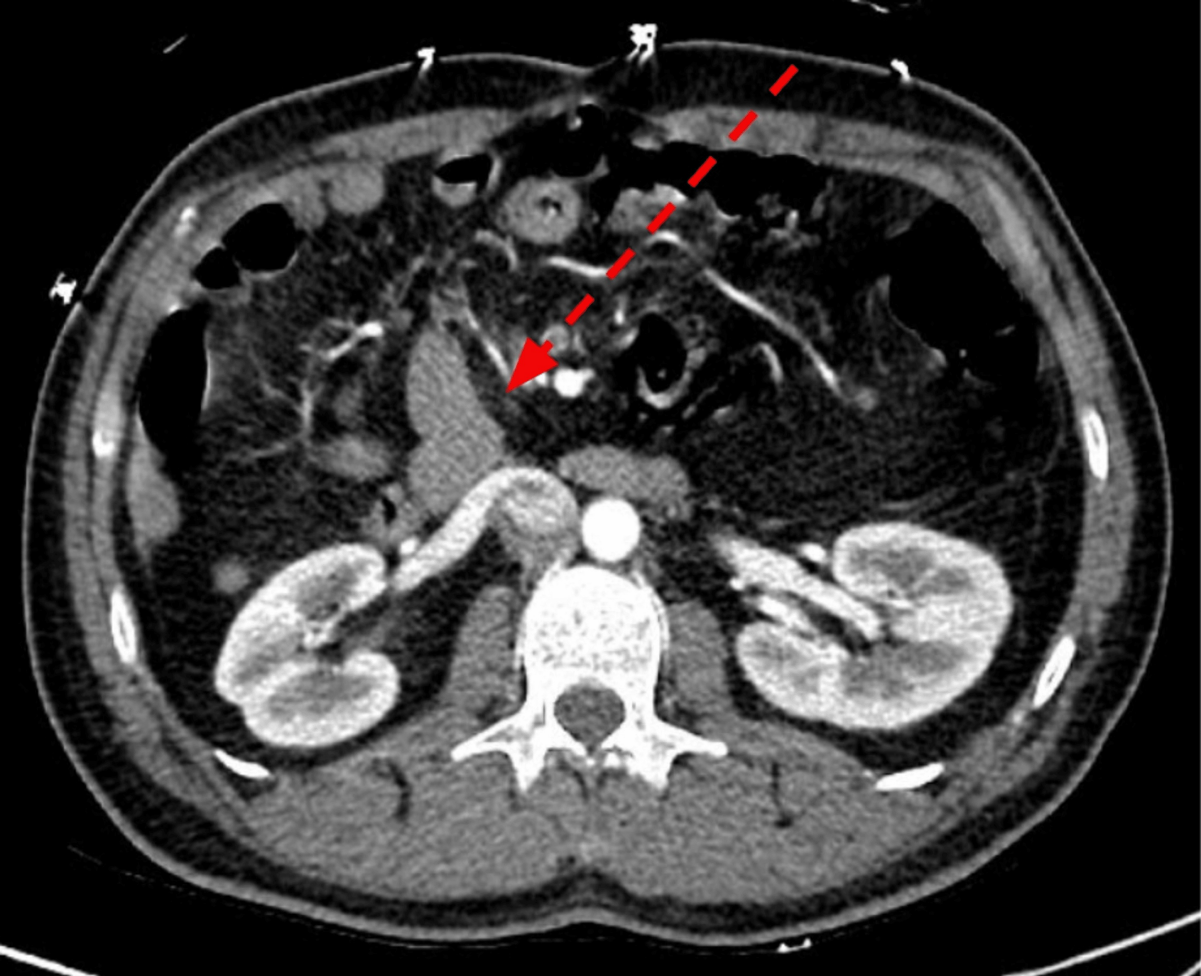
Slightly more inferior to Figure 3, an axial contrast-enhanced CT images at the level of the duodenum. The duodenum (dashed arrow) remains to the right of midline, compatible with congenital gastrointestinal malrotation as suggested on prior sagittal image (Figure 2). CT: Computed tomography

**Figure 5:**
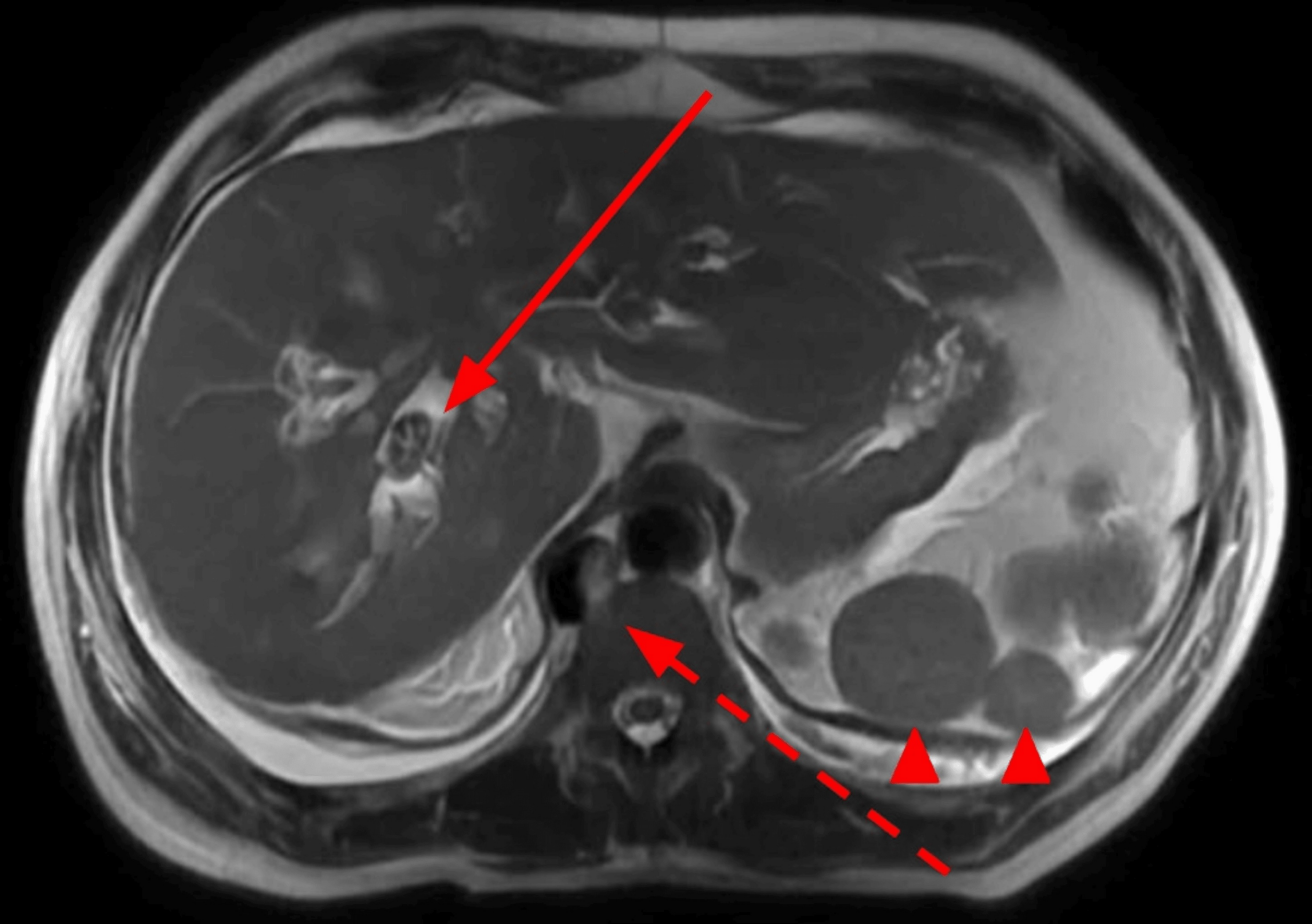
Axial T2-weighted image of the abdomen through the liver demonstrates intrahepatic biliary stone (solid arrow). Redemonstrated is a truncated IVC/absent intrahepatic IVC (dashed arrow) and polysplenia (arrowheads). IVC: Inferior vena cava

**Figure 6:**
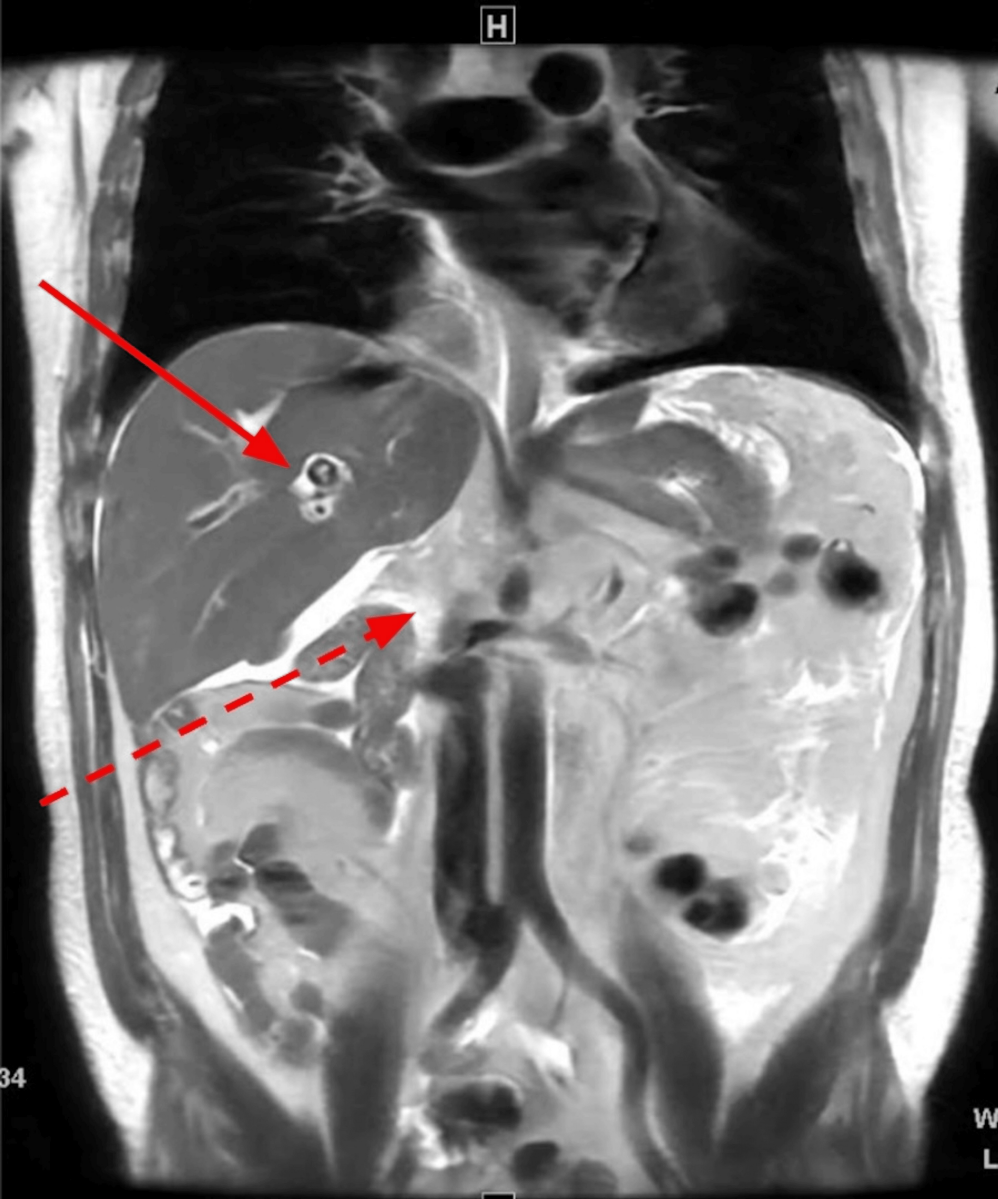
Coronal T2-weighted image of the abdomen through the liver demonstrates intrahepatic biliary stone (solid arrow) and redemonstrated truncated IVC/absent intrahepatic IVC (dashed arrow). IVC: Inferior vena cava

Further imaging with MRIs of the abdomen and liver with and without contrast and an endoscopic retrograde cholangiopancreatography (ERCP) revealed several unusual findings consistent with HS with left isomerism, an incidental yet significant diagnosis. These findings included congenital malrotation without volvulus, bilateral hyparterial bronchi, reversal of the superior mesenteric artery/vein (SMA/V) relationship, a predominantly left-sided large bowel with a right-sided small bowel, interruption of the inferior vena cava (IVC) with azygos continuation, and a truncated pancreas suggestive of dorsal pancreas agenesis. Additional findings relevant to the patient’s acute presentation include a large intrahepatic biliary stone (hepatolithiasis) measuring 1.6 cm, biliary dilatation and segmental hepatic hyperemia highly suggestive of cholangitis, pneumobilia, and mild morphologic changes suggestive of chronic liver disease (Figures 1-6).

The patient's condition deteriorated in the ED, leading to septic shock, for which he received aggressive fluid resuscitation, vasopressor support, and broad-spectrum antibiotics. Blood cultures grew *Citrobacter koseri.* Following admission to the ICU, an ERCP identified a giant stone in the left hepatic duct that could not be removed, necessitating stent placement. Repeat ERCP with lithotripsy successfully cleared the stone burden. The patient stabilized hemodynamically post-intervention and was subsequently transferred to a step-down unit. After six days in the hospital, he was discharged with oral antibiotics, and instructions to follow up with his PCP and a gastroenterologist in one to two weeks. Unfortunately, the patient was later lost to follow-up.

## Discussion

HS is a complex congenital disorder characterized by abnormal arrangement of thoracic and abdominal organs along the left-right axis. Patients with HS fall within a wide spectrum of presentations and severity in their condition depending on organ involvement [5,6]. The spectrum of HS, encompassing both left and right isomerism, presents a diagnostic challenge due to the variability in organ involvement and associated anomalies. This variability complicates not only the classification but also the management of patients with HS, especially when they have not previously been diagnosed with HS and present with acute sequelae, as in this case.

HS follows various inheritance patterns, including autosomal dominant, autosomal recessive, and X-linked recessive, which may partly explain its higher prevalence in males [7]. However, most evidence points to a multifactorial inheritance [8]. The exact timing is unclear, but most abnormalities in asplenia syndrome can be traced back to around 28 days of gestation [9,10]. During this period, the primitive heart and venous connections begin to form. Disruptions at this early stage, when the heart chambers are still developing, may explain common atrial, ventricular, and vascular anomalies in HS [4]. Hutchins et al. suggested that minor changes in the body shape of the embryo could explain the range of anatomical variations in HS [11]. This condition likely results from a primary defect in lateralization, leading to the failure of normal asymmetric development. Although early studies treated asplenia and polysplenia as distinct conditions, later research indicated they were part of a single spectrum [7,12]. Based on Al-Talalwah and Soames' axial sciatic theory, the variability of neurovascular courses is due to the vascular demand of tissue during development. Therefore, congenital anomalies may change the vascular demand requirement, resulting in vascular variation that radiologists and surgeons must be aware of to prevent iatrogenic errors [13].

The incidence of congenital heart disease (CHD) in patients with HS is as high as 50-100% [4]. Right isomerism is associated with more severe cardiac features and thus has a worse prognosis when compared to those with left isomerism. Notably, our patient with left isomerism was not determined to have any cardiac anomalies. Patients with polysplenia syndrome who survive to adulthood are often diagnosed incidentally on CT or MRI during the evaluation of other medical complaints [14]. The 1-year mortality rate for asplenia and polysplenia is >85 and >50%, respectively [1]. A normal splenic function is not necessarily maintained in patients with polysplenia. Therefore, patients with compromised splenic function are at an increased risk of infection and mortality. Those with asplenia tend to have a higher risk of infection, but data on this is limited [6,15].

In this case, the patient's left isomerism manifested with several hallmark features of left isomerism, including congenital malrotation without volvulus, bilateral hyparterial bronchi, reversal of the SMA/V relationship, a predominantly left-sided large bowel with a right-sided small bowel, interruption of the IVC with azygos continuation, and a truncated pancreas suggestive of dorsal pancreas agenesis. Table 1 shows the findings consistent with left isomerism, yet the features are often clinically silent and may only become apparent incidentally during imaging for other conditions [5,14]. The incidental discovery of such significant congenital anomalies underscores the importance of considering HS in patients with atypical or unexplained anatomical findings on imaging, particularly when they present with acute abdominal or systemic symptoms.

**Table 1:** Characteristic differences between left and right isomerism IVC: Inferior vena cava; CHD: Congenital heart disease

Left Isomerism	Right Isomerism
May present in infancy or adulthood	Most present in infancy/neonatal period
Polysplenia	Asplenia
Bilobed lungs, hyparterial bronchi	Trilobed lungs, eparterial bronchi
Interrupted IVC with azygous continuation	Cyanotic CHD, bilateral right atria
Better prognosis	Worse prognosis

The presence of hepatolithiasis in this patient with HS and left isomerism complicates the clinical picture, as the condition is not typically associated with HS. There is only one other reported case of HS with acute cholangitis and another case of HS with hepatolithiasis [16,17]. It is suspected that anomalies related to the portal vein, pancreas, or intestinal malrotation may predispose these patients to obstruction by hindering the flow of bile, thus leading to pathologies such as cholangitis, pancreatitis, choledocolithiasis, or hepatolithiasis [17]. However, the combination of biliary anomalies, such as pneumobilia and segmental hepatic hyperemia, as well as a history of cholecystectomy, suggests a predisposition to biliary stasis and stone formation in this patient, potentially exacerbated by his underlying congenital anomalies.

The patient's deterioration into septic shock highlights the potential for rapid clinical decline in patients with HS, particularly when complicated by infections. The identification of *C. koseri*, a rare but recognized pathogen in cholangitis, further complicates the case emphasizing the need for aggressive and targeted treatment in similar presentations [18].

This case also raises important considerations regarding managing HS patients often diagnosed incidentally during adulthood. Given the potential for complex anatomical and functional anomalies, a multidisciplinary approach involving radiologists, gastroenterologists, and surgeons is essential for optimizing outcomes in these patients. Patients with HS are often managed symptomatically and surgery is often performed to relieve biliary or intestinal obstructions [19,20]. Consequently, patients undergoing abdominal surgery are at increased risk of complications due to their unusual anatomy [16]. Additionally, the long-term follow-up of such patients is important, as they may be at increased risk for recurrent infections, biliary complications, and other sequelae related to their underlying congenital abnormalities.

## Conclusions

This case illustrates the complexity and clinical challenges associated with HS with left isomerism, particularly when complicated by hepatolithiasis, pneumobilia, and acute cholangitis. The incidental diagnosis of these congenital anomalies in a middle-aged patient underscores the critical role of comprehensive imaging and the need for a high index of suspicion in patients with atypical presentations. The case also highlights the importance of a multidisciplinary approach in managing such patients, who may present with a wide spectrum of clinical issues that require coordinated care. Future research should focus on better understanding the long-term outcomes and optimal management strategies for patients with HS diagnosed in adulthood, particularly those with left isomerism and associated gastrointestinal anomalies.
